# Results from the Survey of Antibiotic Resistance (SOAR) 2020–21 in Vietnam: data based on CLSI, EUCAST (dose-specific) and pharmacokinetic/pharmacodynamic (PK/PD) breakpoints

**DOI:** 10.1093/jac/dkaf290

**Published:** 2025-11-24

**Authors:** Didem Torumkuney, Pham Hung Van, Le Quoc Thinh, Stephen Hawser, Subhashri Kundu, Ngoc Truong Ha Lan, Anand Manoharan

**Affiliations:** Infectious Diseases Research Unit, GSK, London, UK; Department of Microbiology, Vietnam Research and Development Institute of Clinical Microbiology (VCM), Ho Chi Minh City, Vietnam; Pediatric Department, Pediatric 1 Hospital, Ho Chi Minh City, Vietnam; Global Affairs, IHMA Europe Sàrl, Monthey, Switzerland; Infectious Diseases - Antibiotic Resistance, GSK, Singapore, Republic of Singapore; Vaccine Department, GSK, Ho Chi Minh City, Vietnam; Infectious Diseases Medical & Scientific Affairs, GSK, Mumbai, India

## Abstract

**Objectives:**

Antibiotic susceptibility determination of *Streptococcus pneumoniae* and *Haemophilus influenzae* from community-acquired respiratory infections collected in 2020–21 from two hospitals in Ho Chi Minh City, Vietnam.

**Methods:**

MICs were determined by CLSI broth microdilution; susceptibility was interpreted based on CLSI, EUCAST and pharmacokinetic/pharmacodynamic (PK/PD) breakpoints.

**Results:**

A total of 144 *S. pneumoniae* and 191 *H. influenzae* isolates were collected. *S. pneumoniae* susceptibility to amoxicillin and amoxicillin/clavulanic acid (2:1) was 56.9% and 56.3% (CLSI), 29.2% and 25.7% (EUCAST high-dose) and 78.5% using high-dose amoxicillin (4 g/0.25 g/day, PK/PD). Susceptibility to ceftriaxone was 91.7% by EUCAST (high-dose) and 50.7% by CLSI or PK/PD. Pneumococcal cefotaxime susceptibility was 61.8% by CLSI (29.9%, EUCAST). Penicillin susceptibility was 69.4% by CLSI intravenous/EUCAST high-dose, but 6.3% using CLSI oral/EUCAST low-dose breakpoints. Tetracyclines, macrolides and trimethoprim/sulfamethoxazole had 5.6%–16.7% susceptibility.

Among *H. influenzae* isolates, 59.7% were β-lactamase-negative, of which 87.7% and 74.6% were ampicillin-resistant (BLNAR) following EUCAST and CLSI criteria, respectively. *H. influenzae* fluoroquinolone, macrolide and tetracycline susceptibility reached 62.0%–89.5% (CLSI, EUCAST); macrolide susceptibility was 3.7%–5.8% (PK/PD). All were susceptible to ceftriaxone (all breakpoints) and cefotaxime (CLSI, PK/PD); cefotaxime susceptibility was 15.7% by EUCAST. *H. influenzae* amoxicillin/clavulanic acid susceptibility was 45.5% (EUCAST) and 32.5% (CLSI) but increased to 82.8% with high-dose (4 g/0.25 g/day, PK/PD).

**Conclusions:**

The hospitals showed high rates of penicillin-resistant *S. pneumoniae* and BLNAR *H. influenzae*. *S. pneumoniae* showed low susceptibility to macrolides and several cephalosporins. *H. influenzae* fluoroquinolone susceptibility was 88.0%–89.0% (CLSI). Future surveillance across Vietnam will help understand susceptibility trends over time and regions.

## Introduction

Despite a substantial reduction since 1990, community-acquired pneumonia and other lower respiratory tract infections (RTIs) still rank as the second most frequent cause of all-age premature deaths at the global level.^1^  *Streptococcus pneumoniae* and *Haemophilus influenzae* are the major bacteria associated with community-acquired RTIs (CA-RTIs),^2,3^ and both have shown increasing resistance to first-line antibiotics such as penicillin and ampicillin.^4,5^ Since rates of resistance vary over time and differ between countries,^6^ up-to-date surveillance data are essential to guide local antibiotic policies.

The Survey of Antibiotic Resistance (SOAR) represents a pioneering and extensive antimicrobial resistance (AMR) surveillance initiative that has been operational since 2002.^7^ SOAR focuses on the assessment of AMR in key respiratory pathogens, notably *S. pneumoniae* and *H. influenzae*, across a vast spectrum of geographic areas, encompassing the Middle East, Africa, Latin America, Asia-Pacific, Europe and the Commonwealth of Independent States countries.

In a previous SOAR study with data from the same two hospitals in Vietnam conducted during 2016–18, a high level of penicillin and macrolide resistance among *S. pneumoniae* was noted.^8^ Here, we report recent data from SOAR for *S. pneumoniae* and *H. influenzae* recovered from CA-RTIs at two hospital sites in Ho Chi Minh City, Vietnam, during the period 2020–21.

## Materials and methods

### Ethics

SOAR studies are not human subject studies. During the study, only microorganisms were examined.

### Collaborating centres

Isolates were provided between 2020 and 2021 from two sites in Ho Chi Minh City, Vietnam: Nguyen Tri Phuong Hospital and Pediatric 1 Hospital.

### Clinical isolates

Isolates of *H. influenzae* and *S. pneumoniae* from CA-RTIs (isolated within 48 h of hospitalization) were sent to a central laboratory [International Health Management Associates (IHMA) Europe, Monthey, Switzerland], to be subcultured and reidentified. *H. influenzae* were reidentified by MALDI-TOF MS methodology; *S. pneumoniae* identity was confirmed by optochin susceptibility and bile solubility. For each *H. influenzae*, isolate β-lactamase production was determined by a chromogenic cephalosporin (nitrocefin) disc method. Duplicate isolates from the same patient were not accepted.

### Susceptibility testing

Broth microdilution methodology (recommended by CLSI) was used to evaluate isolates for antibiotic susceptibility.^9^ Amoxicillin, amoxicillin/clavulanic acid (2:1 ratio as per CLSI guidelines),^10^ amoxicillin/clavulanic acid (fixed clavulanic acid at 2 mg/L as per EUCAST guidelines),^11^ azithromycin, cefaclor, cefdinir, cefixime, cefotaxime, cefpodoxime, ceftibuten, ceftriaxone, cefuroxime, clarithromycin, levofloxacin, moxifloxacin, tetracycline and trimethoprim/sulfamethoxazole (1:19 ratio) were tested against both *S. pneumoniae* and *H. influenzae*. Doxycycline, erythromycin and penicillin were tested against *S. pneumoniae* only; ampicillin was tested against *H. influenzae* only. Susceptibility to the study drugs was calculated based on CLSI, EUCAST (dose-specific) and pharmacokinetic/pharmacodynamic (PK/PD) breakpoints;^10–12^ these breakpoints are shown in Tables 1–3. The analyses included susceptibility rates based on a higher amoxicillin dosage of 4 g/day and a higher dosage of 4 g amoxicillin/0.25 g clavulanic acid per day [using amoxicillin/clavulanic acid (2:1) MICs] according to a published PK/PD susceptible breakpoint of ≤4 mg/L (amoxicillin component).^12^

**Table 1.** CLSI MIC breakpoints (mg/L) used for *S. pneumoniae* and *H. influenzae* isolates

Antimicrobial	*S. pneumoniae*			*H. influenzae*		
	S	I	R	S	I	R
Amoxicillin	≤2	4	≥8	—	—	—
Amoxicillin/clavulanic acid (2:1)^a^	≤2	4	≥8	≤2	4	≥8
Ampicillin	NT	NT	NT	≤1	2	≥4
Azithromycin	≤0.5	1	≥2	≤4	—	—
Cefaclor	≤1	2	≥4	≤8	16	≥32
Cefdinir	≤0.5	1	≥2	≤1	—	—
Cefixime	—	—	—	≤1	—	—
Cefotaxime (non-meningitis)	≤1	2	≥4	≤2	—	—
Cefpodoxime	≤0.5	1	≥2	≤2	—	—
Ceftibuten	—	—	—	≤2	—	—
Ceftriaxone (non-meningitis)	≤1	2	≥4	≤2	—	—
Cefuroxime^b^	≤1	2	≥4	≤4	8	≥16
Clarithromycin	≤0.25	0.5	≥1	≤8	16	≥32
Doxycycline	≤0.25	0.5	≥1	NT	NT	NT
Erythromycin	≤0.25	0.5	≥1	NT	NT	NT
Levofloxacin	≤2	4	≥8	≤2	—	—
Moxifloxacin	≤1	2	≥4	≤1	—	—
Penicillin (2.4 g 2 MU × 4–6 IV)	≤2	4	≥8	NT	NT	NT
Penicillin (oral)	≤0.06	0.12–1	≥2	NT	NT	NT
Tetracycline	≤1	2	≥4	≤2	4	≥8
Trimethoprim/sulfamethoxazole^c^	≤0.5	1–2	≥4	≤0.5	1–2	≥4

—, not applicable; I, intermediate; NT, not tested; R, resistant; S, susceptible.

^a^Amoxicillin/clavulanic acid was tested at a 2:1 amoxicillin to clavulanic acid ratio; breakpoints are expressed as the amoxicillin component.

^b^Breakpoints used are for cefuroxime axetil (oral).

^c^Trimethoprim/sulfamethoxazole was tested at a 1:19 trimethoprim to sulfamethoxazole ratio; breakpoints are expressed as the trimethoprim component.

**Table 2.** EUCAST (dose-specific) MIC breakpoints (mg/L) used for *S. pneumoniae* and *H. influenzae* isolates

Antimicrobial^a^	*S. pneumoniae*		*H. influenzae*	
	S	R	S	R
Amoxicillin (0.5 g × 3 oral)	≤0.5	>1	≤0.001	>2
Amoxicillin (0.75–1 g × 3 oral)	≤1	>1	≤2	>2
Amoxicillin/clavulanic acid (0.5 g/0.125 g × 3 oral)^b^	≤0.5	>1	≤0.001	>2
Amoxicillin/clavulanic acid (0.875 g/0.125 g × 3 oral)^b^	≤1	>1	≤2	>2
Ampicillin (2 g × 3 IV)	NT	NT	≤1	>1
Ampicillin (2 g × 4 IV)	NT	NT	≤1	>1
Azithromycin	≤0.25	>0.5	—	—
Cefaclor	≤0.001	>0.5	—	—
Cefdinir	—	—	—	—
Cefixime	—	—	≤0.12	>0.12
Cefotaxime	≤0.5	>2	≤0.12	>0.12
Cefpodoxime	≤0.25	>0.5	≤0.25	>0.25
Ceftibuten	—	—	≤1	>1
Ceftriaxone (1 g × 1 IV)	≤0.5	>2	≤0.12	>0.12
Ceftriaxone (2 g × 2 IV)	≤2	>2	≤0.12	>0.12
Cefuroxime^c^	≤0.25	>0.5	≤0.001	>1
Clarithromycin (0.25 g × 2 oral)	≤0.25	>0.5	—	—
Clarithromycin (0.5 g × 2 oral)	≤0.5	>0.5	—	—
Doxycycline	≤1	>2	NT	NT
Erythromycin (0.5 g × 2–4 oral or 0.5 g × 2–4 IV)	≤0.25	>0.5	NT	NT
Erythromycin (1 g × 4 oral or 1 g × 4 IV)	≤0.5	>0.5	NT	NT
Levofloxacin (0.5 g × 2 oral or 0.4 g × 2 IV)	≤0.001	>2	≤0.06	>0.06
Levofloxacin (0.75 g × 2 oral or 0.4 g × 3 IV)	≤2	>2	≤0.06	>0.06
Moxifloxacin	≤0.5	>0.5	≤0.12	>0.12
Penicillin (0.6 g 1 MU × 4 IV)	≤0.06	>2	NT	NT
Penicillin (2.4 g 2 MU × 4–6 IV)	≤2	>2	NT	NT
Tetracycline	≤1	>2	≤2	>2
Trimethoprim/sulfamethoxazole (0.16 g/0.8 g × 2 oral or IV)^d^	≤1	>2	≤0.5	>1
Trimethoprim/sulfamethoxazole (0.24 g/1.2 g × 2 oral or IV)^d^	≤2	>2	≤1	>1

—, not applicable; NT, not tested; R, resistant; S, susceptible.

^a^Where available, susceptibility was assessed using EUCAST higher dosage breakpoints.

^b^Amoxicillin/clavulanic acid was tested at a fixed concentration of 2 mg/L; breakpoints are expressed as the amoxicillin component.

^c^Breakpoints used are for cefuroxime axetil (oral).

^d^Trimethoprim/sulfamethoxazole was tested at a 1:19 trimethoprim to sulfamethoxazole ratio; breakpoints are expressed as the trimethoprim component.

**Table 3.** PK/PD MIC breakpoints (mg/L) used for *S. pneumoniae* and *H. influenzae* isolates

Antimicrobial	*S. pneumoniae* and *H. influenzae*
	S only
Amoxicillin (1.5 g/day)^a^	≤2
Amoxicillin (4 g/day)^b^	≤4
Amoxicillin/clavulanic acid^a^ (1.75 g/0.25 g/day adults; 45 g/6.4 mg/kg/day children)	≤2
Amoxicillin/clavulanic acid^b^ (4 g/0.25 g/day adults; 90 mg/6.4 mg/kg/day children)	≤4
Ampicillin	—
Penicillin	—
Cefaclor	≤0.5
Cefdinir	≤0.25
Cefditoren	—
Cefixime	≤1
Cefpodoxime	≤0.5
Ceftriaxone	≤1
Cefuroxime^c^	≤1
Azithromycin	≤0.12
Clarithromycin	≤0.25
Erythromycin	≤0.25
Levofloxacin	≤2
Moxifloxacin	≤1
Trimethoprim/sulfamethoxazole^d^	≤0.5

—, not applicable; PK/PD, pharmacokinetic/pharmacodynamic; S, susceptible.

^a^Amoxicillin/clavulanic acid for low dose in adults/children.

^b^Amoxicillin/clavulanic acid for high dose in adults/children.

^c^Breakpoints used are for cefuroxime axetil (oral).

^d^Trimethoprim/sulfamethoxazole was tested at a 1:19 trimethoprim to sulfamethoxazole ratio; breakpoints are expressed as the trimethoprim component.

### Quality control and data analysis

On each day of testing, quality control strains *S. pneumoniae* ATCC 49619, *H. influenzae* ATCC 49247, *H. influenzae* ATCC 49766 and *Escherichia coli* ATCC 32518 were included. Results of susceptibility testing were only accepted if the quality control strain results were within the published acceptable range. Fisher’s exact test (using XLSTAT version 2023.1.1.1399) was used to assess statistical significance in differences in susceptibility (using CLSI criteria) across penicillin-susceptible isolates (*S. pneumoniae* only). A *P* value <0.05 was considered statistically significant.

## Results

### *S. pneumoniae* isolates

A total of 144 clinical isolates of *S. pneumoniae* collected from two hospitals in Ho Chi Minh City, Vietnam, in 2020–21 were analysed. The source of the *S. pneumoniae* isolates included sputum (*n* = 85, 59.0%), endotracheal aspirate (*n* = 31, 21.5%) and blood (*n* = 21, 14.6%). Smaller numbers of isolates were from middle ear (*n* = 3, 2.1%), sinuses (*n* = 2, 1.4%) and other respiratory specimens (*n* = 2, 1.4%). Overall, 46.5% (*n* = 67) came from adolescents and adults (13–64 years), 8.3% (*n* = 12) from elderly (≥65 years) and 45.1% (*n* = 65) from paediatric patients (aged ≤12 years).

Summary MIC and susceptibility data for all *S. pneumoniae* are shown in Tables 4–6. MIC distribution data are provided in Table S1 (available as Supplementary data at *JAC* Online).

**Table 4.** Summary MIC and susceptibility data for *S. pneumoniae* (*n* = 144) from Vietnam using CLSI breakpoints

Antimicrobial	MIC (mg/L)			CLSI susceptibility		
	Range	50%	90%	%S	%I	%R
Amoxicillin	0.015 to >8	2	8	56.9	21.5	21.5
Amoxicillin/clavulanic acid (2:1)	≤0.008 to >8	2	8	56.3	22.2	21.5
Penicillin (2.4 g 2 MU × 4–6 IV)	0.015–8	0.12	0.12	69.4	28.5	2.1
Penicillin (oral)	0.015–8	2	4	6.3	26.4	67.4
Cefaclor	0.03 to >4	>4	>4	10.4	4.2	85.4
Cefdinir	≤0.015 to >8	4	>8	18.1	2.1	79.9
Cefixime	≤0.25 to >16	16	>16	—	—	—
Cefotaxime	≤0.008 to >4	1	2	61.8	31.3	6.9
Cefpodoxime	≤0.015 to >4	2	>4	19.4	10.4	70.1
Ceftibuten	≤0.5 to >16	>16	>16	—	—	—
Ceftriaxone	0.015 to >4	1	2	50.7	41.0	8.3
Cefuroxime	≤0.008 to >8	4	>8	16.0	11.1	72.9
Azithromycin	≤0.015 to >16	>16	>16	6.9	0.7	92.4
Clarithromycin	≤0.015 to >16	>16	>16	7.6	0.7	91.7
Erythromycin	≤0.015 to >16	>16	>16	5.6	0.7	93.8
Doxycycline	≤0.008 to >4	4	>4	13.2	1.4	85.4
Tetracycline	≤0.03 to >4	>4	>4	13.9	0.7	85.4
Levofloxacin	≤0.12 to >8	1	1	95.8	0	4.2
Moxifloxacin	≤0.03 to >4	0.12	0.12	95.1	2.1	2.8
Trimethoprim/sulfamethoxazole	≤0.06 to >8	4	>8	15.3	7.6	77.1

—, not applicable; I, intermediate; R, resistant; S, susceptible.

**Table 5.** Summary MIC and susceptibility data for *S. pneumoniae* (*n* = 144) from Vietnam using EUCAST (dose-specific) breakpoints

Antimicrobial	MIC (mg/L)			EUCAST susceptibility		
	Range	50%	90%	%S	%I	%R
Amoxicillin (0.5 g × 3 oral)	0.015 to >8	2	8	18.1	11.1	70.8
Amoxicillin (0.75–1 g × 3 oral)	0.015 to >8	2	8	29.2	—	70.8
Amoxicillin/clavulanic acid (0.5 g/0.125 g × 3 oral)	≤0.008 to >8	2	8	16.7	9.0	74.3
Amoxicillin/clavulanic acid (0.875 g/0.125 g × 3 oral)	≤0.008 to >8	2	8	25.7	—	74.3
Penicillin (0.6 g 1 MU × 4 IV)	0.015–8	0.12	0.12	6.3	63.2	30.6
Penicillin (2.4 g 2 MU × 4–6 IV)	0.015–8	0.12	0.12	69.4	—	30.6
Cefaclor	0.03 to >4	>4	>4	0	6.3	93.8
Cefdinir	≤0.015 to >8	4	>8	—	—	—
Cefixime	≤0.25 to >16	16	>16	—	—	—
Cefotaxime	≤0.008 to >4	1	2	29.9	63.2	6.9
Cefpodoxime	≤0.015 to >4	2	>4	13.9	5.6	80.6
Ceftibuten	≤0.5 to >16	>16	>16	—	—	—
Ceftriaxone (1 g × 1 IV)	0.015 to >4	1	2	24.3	67.4	8.3
Ceftriaxone (2 g × 2 IV)	0.015 to >4	1	2	91.7	—	8.3
Cefuroxime	≤0.008 to >8	4	>8	11.1	1.4	87.5
Azithromycin	≤0.015 to >16	>16	>16	6.3	0.7	93.1
Clarithromycin (0.25 g × 2 oral)	≤0.015 to >16	>16	>16	7.6	0.7	91.7
Clarithromycin (0.5 g × 2 oral)	≤0.015 to >16	>16	>16	8.3	—	91.7
Erythromycin (0.5 g × 2–4 oral or 0.5 g × 2–4 IV)	≤0.015 to >16	>16	>16	5.6	0.7	93.8
Erythromycin (1 g × 4 oral or 1 g × 4 IV)	≤0.015 to >16	>16	>16	6.3	—	93.8
Doxycycline	≤0.008 to >4	4	>4	21.5	12.5	66.0
Tetracycline	≤0.03 to >4	>4	>4	13.9	0.7	85.4
Levofloxacin (0.5 g × 2 oral or 0.4 g × 2 IV)	≤0.12 to >8	1	1	0	95.8	4.2
Levofloxacin (0.75 g × 2 oral or 0.4 g × 3 IV)	≤0.12 to >8	1	1	95.8	—	4.2
Moxifloxacin	≤0.03 to >4	0.12	0.12	95.1	—	4.9
Trimethoprim/sulfamethoxazole (0.16 g/0.8 g × 2 oral or IV)	≤0.06 to >8	4	>8	16.7	6.3	77.1
Trimethoprim/sulfamethoxazole (0.24 g/1.2 g × 2 oral or IV)	≤0.06 to >8	4	>8	22.9	—	77.1

—, not applicable; I, susceptible, increased exposure; R, resistant; S, susceptible.

**Table 6.** Summary MIC and susceptibility data for *S. pneumoniae* (*n* = 144) from Vietnam using PK/PD breakpoints

	MIC (mg/L)			PK/PD susceptibility
Antimicrobial	Range	50%	90%	%S
Amoxicillin (1.5 g/day)	0.015 to >8	2	8	56.9
Amoxicillin (4 g/day)	0.015 to >8	2	8	78.5
Amoxicillin/clavulanic acid (1.75 g/0.25 g/day adults; 45 mg/6.4 mg/kg/day children)	≤0.008 to >8	2	8	56.3
Amoxicillin/clavulanic acid (4 g/0.25 g/day adults; 90 mg/6.4 mg/kg/day children)	≤0.008 to >8	2	8	78.5
Penicillin	0.015–8	0.12	0.12	—
Cefaclor	0.03 to >4	>4	>4	6.3
Cefdinir	≤0.015 to >8	4	>8	12.5
Cefixime	≤0.25 to >16	16	>16	13.9
Cefotaxime	≤0.008 to >4	1	2	—
Cefpodoxime	≤0.015 to >4	2	>4	19.4
Ceftibuten	≤0.5 to >16	>16	>16	—
Ceftriaxone	0.015 to >4	1	2	50.7
Cefuroxime	≤0.008 to >8	4	>8	16.0
Azithromycin	≤0.015 to >16	>16	>16	5.6
Clarithromycin	≤0.015 to >16	>16	>16	7.6
Erythromycin	≤0.015 to >16	>16	>16	5.6
Doxycycline	≤0.008 to >4	4	>4	13.2
Tetracycline	≤0.03 to >4	>4	>4	—
Levofloxacin	≤0.12 to >8	1	1	95.8
Moxifloxacin	≤0.03 to >4	0.12	0.12	95.1
Trimethoprim/sulfamethoxazole	≤0.06 to >8	4	>8	15.3

—, not applicable; PK/PD, pharmacokinetic/pharmacodynamic; S, susceptible.

### *S. pneumoniae* susceptibility

When applying CLSI, EUCAST or PK/PD breakpoints, levofloxacin and moxifloxacin showed 95.1%–95.8% susceptibility among tested antimicrobials. By CLSI oral or EUCAST low-dose breakpoints, only 6.3% of the pneumococci were penicillin-susceptible, 26.4% were intermediate and 67.4% were resistant by CLSI, whereas 63.2% were susceptible, with increased exposure (former intermediate category), and 30.6% were resistant according to EUCAST. With CLSI intravenous (IV) or EUCAST high-dose (2.4 g × 6 IV) penicillin breakpoints, susceptibility increased to 69.4% (*n* = 100).

By applying CLSI breakpoints, overall resistance rates of 21.5% were observed for amoxicillin and for amoxicillin/clavulanic acid (2:1). Amoxicillin and amoxicillin/clavulanic acid (2:1) showed susceptibility of 56.9% and 56.3% by CLSI criteria, respectively. When applying EUCAST breakpoints, the rate of resistant pneumococci was 70.8% for amoxicillin (0.75–1 g × 3 oral) and 74.3% for amoxicillin/clavulanic acid (2 mg/L) (0.875 g/0.125 g × 3 oral). However, higher-dose amoxicillin/clavulanic acid (4 g/0.25 g/day) or amoxicillin (4 g/day) increased susceptibility to 78.5% (PK/PD breakpoint).

**Figure 1.**
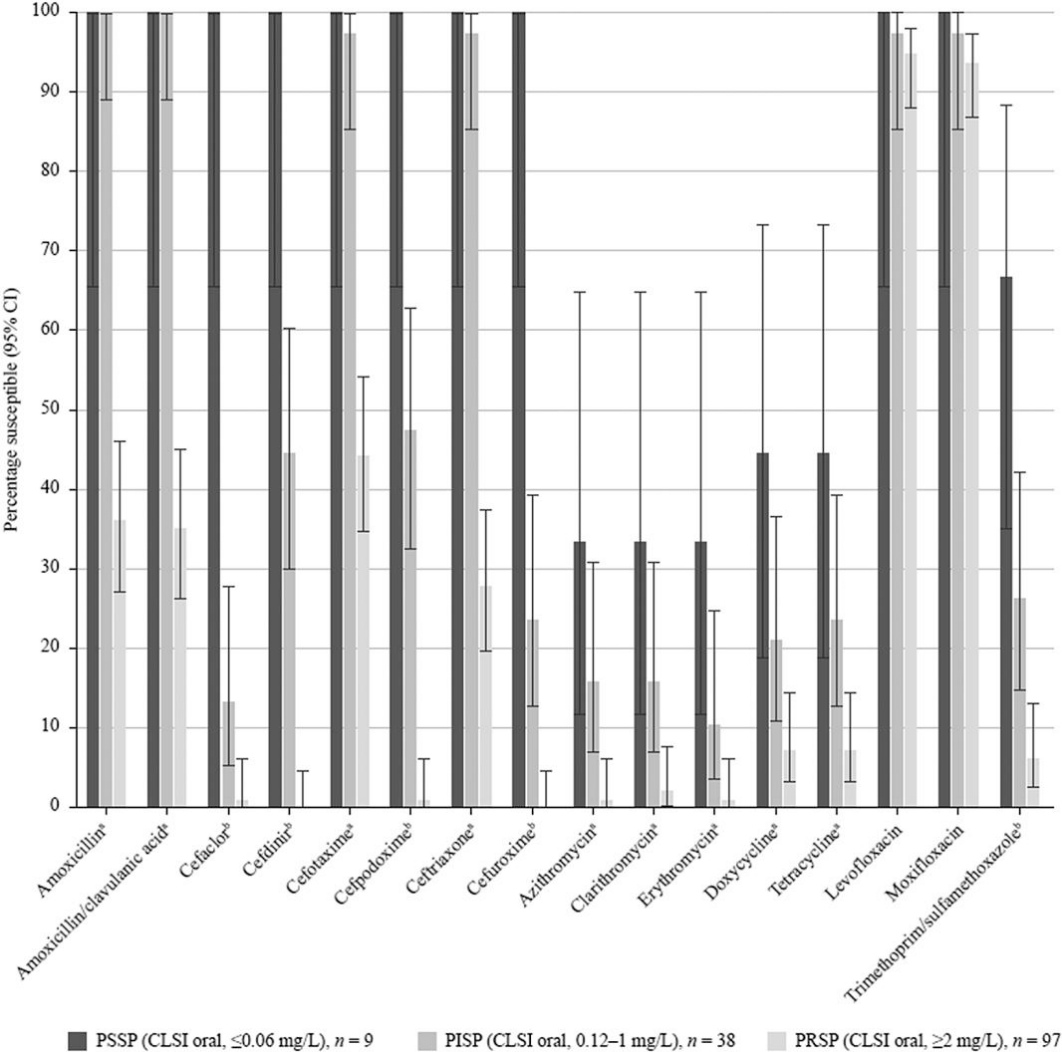
Susceptibility rates (with 95% CI) based on CLSI breakpoints for antibiotics against PSSP, PISP and PRSP from Vietnam. ^a^Statistically significant decrease in susceptibility for PRSP compared with PSSP and for PRSP compared with PISP. Amoxicillin and amoxicillin/clavulanic acid (2:1), *P* = 0.0002 and <0.0001, respectively; cefotaxime, *P* = 0.001 and <0.0001, respectively; ceftriaxone, both *P* < 0.0001; azithromycin, both *P* = 0.002; clarithromycin, *P* = 0.004 and 0.006, respectively; erythromycin, *P* = 0.002 and 0.022, respectively; doxycycline, *P* = 0.006 and 0.032, respectively; tetracycline, *P* = 0.006 and 0.007, respectively. ^b^Statistically significant decrease in susceptibility for PISP compared with PSSP, for PRSP compared with PSSP and for PRSP compared wih PISP. Cefaclor, *P* < 0.0001,  < 0.0001 and 0.007, respectively; cefdinir, *P* = 0.003,  < 0.0001 and <0.0001, respectively; cefpodoxime, *P* = 0.006,  < 0.0001 and <0.0001, respectively; cefuroxime, all *P* < 0.0001; trimethoprim/sulfamethoxazole (1:19), *P* = 0.045,  < 0.0001 and 0.002, respectively. CI, confidence interval; PISP, penicillin-intermediate *S. pneumoniae*; PRSP, penicillin-resistant *S. pneumoniae*; PSSP, penicillin-susceptible *S. pneumoniae*.

**Figure 2.**
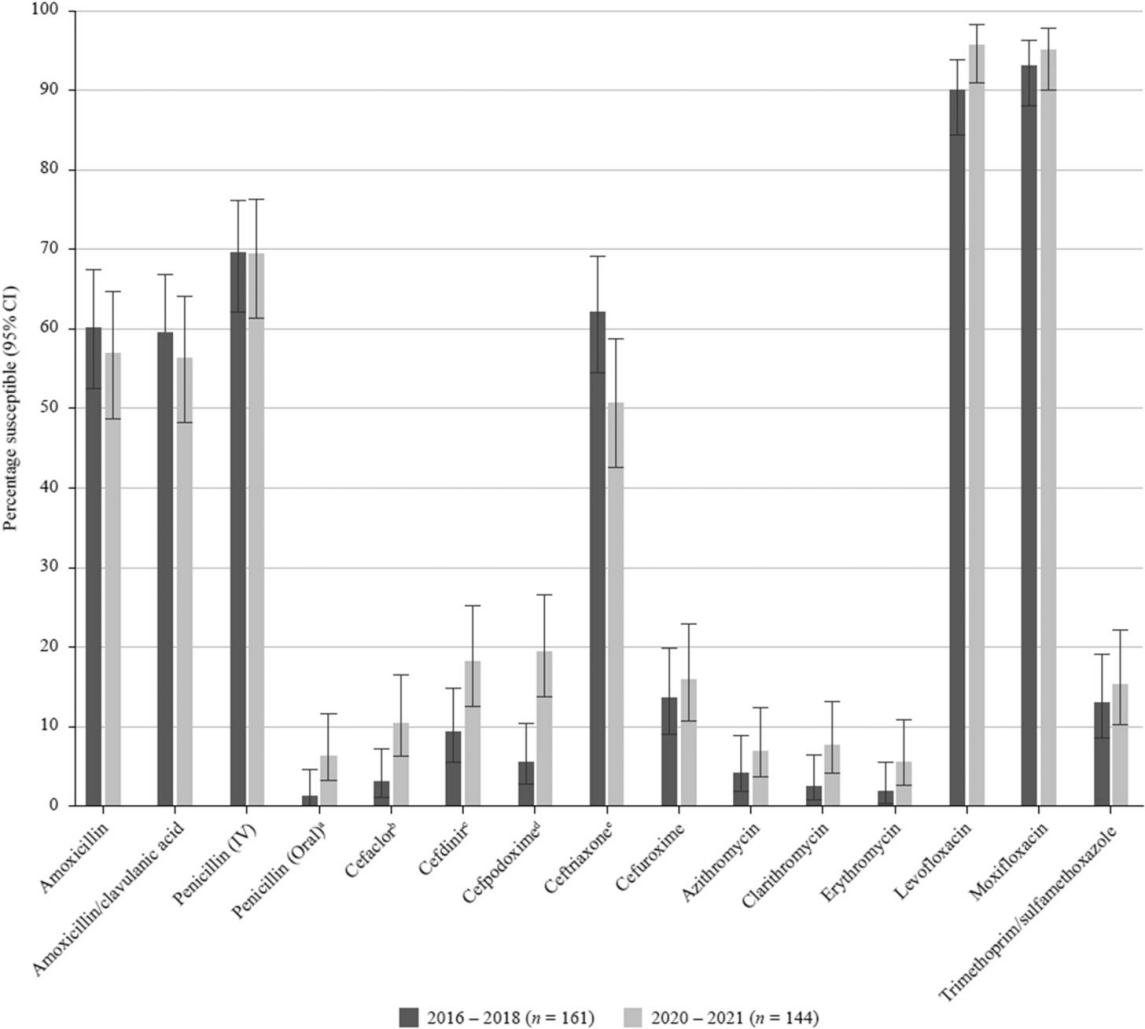
Comparison of antibiotic susceptibility rates of *S. pneumoniae* isolates from Vietnam collected in 2016–18 with isolates collected in 2020–21 (CLSI breakpoints). Statistically significant increase in susceptibility: ^a^*P* = 0.028, ^b^*P* = 0.011, ^c^*P* = 0.029, ^d^*P* = 0.0003. Statistically significant decrease in susceptibility: ^e^*P* = 0.05. CI, confidence interval.

**Figure 3.**
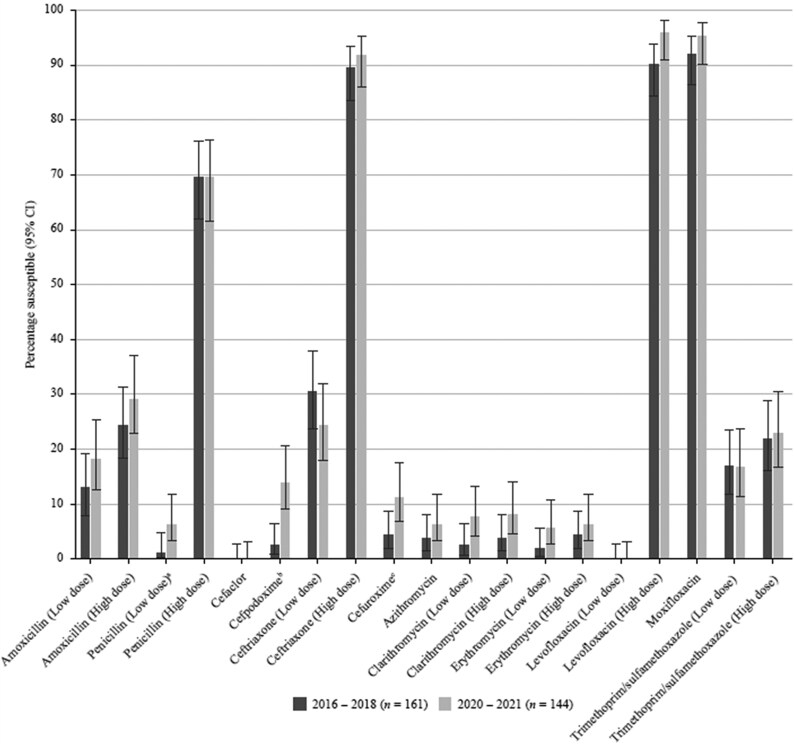
Comparison of antibiotic susceptibility rates of *S. pneumoniae* isolates from Vietnam collected in 2016–18 with isolates collected in 2020–21 (EUCAST breakpoints). Statistically significant increase in susceptibility: ^a^*P* = 0.028, ^b^*P* = 0.0002, ^c^*P* = 0.03. CI, confidence interval.

High resistance level was also seen to cefaclor (CLSI, 85.4%; EUCAST, 93.8%), cefdinir (CLSI, 79.9%; EUCAST, no breakpoints), cefuroxime (CLSI, 72.9%; EUCAST, 87.5%) and cefpodoxime (CLSI, 70.1%; EUCAST, 80.6%). Of the cephalosporins tested, cefotaxime showed a 61.8% overall susceptibility rate by CLSI, although susceptibility was 29.9% by EUCAST. Tetracyclines, macrolides and trimethoprim/sulfamethoxazole were poorly active (5.6%–16.7% susceptibility) with comparable rates between CLSI, EUCAST and PK/PD criteria. Susceptibility to ceftriaxone was 91.7% by the EUCAST high-dose (2 g × 2 IV) breakpoint but reduced to 50.7% by CLSI or PK/PD criteria and to 24.3% by EUCAST low-dose breakpoint. Susceptibility of 0%–19.4% to the other cephalosporins tested was observed with EUCAST as well as with CLSI and PK/PD breakpoints (Tables 4–6 and S1, Figures 1–3).

### Prevalence of antibiotic susceptibility among penicillin-susceptible *S. pneumoniae* (PSSP) and penicillin-resistant *S. pneumoniae* (PRSP) isolates (based on CLSI oral breakpoints)

According to CLSI oral breakpoints (Figure 1), the PSSP isolates were fully susceptible to other β-lactam agents and fluoroquinolones, but poorly susceptible to macrolides (33.3% susceptible), tetracyclines (44.4% susceptible) and trimethoprim/sulfamethoxazole (66.7% susceptible). Penicillin-intermediate *S. pneumoniae* (PISP) and PRSP isolates were statistically less susceptible to cefaclor, cefdinir, cefpodoxime, cefuroxime and trimethoprim/sulfamethoxazole than PSSP isolates. There was no statistically significant difference in the activity of amoxicillin, amoxicillin/clavulanic acid, cefotaxime, ceftriaxone, macrolides, tetracycline and fluoroquinolones against PISP and PSSP isolates; however, all agents except fluoroquinolones were statistically less active against PRSP than PSSP or PISP, demonstrating an association between multidrug resistance and PRSP.

### *H. influenzae* isolates

A total of 191 *H. influenzae* isolates collected from two hospitals in Ho Chi Minh City (South Vietnam) in 2020–21 were analysed. These isolates were mostly from sputum (*n* = 133, 69.6%), endotracheal aspirate (*n* = 39, 20.4%) and bronchoalveolar lavage (*n* = 15, 7.9%). Less frequently, isolates were from blood (*n* = 2, 1.0%), sinuses and middle ear (each *n* = 1, 0.5%). Most isolates of *H. influenzae* (*n* = 106; 55.5%) came from adolescent and adult patients (13–64 years); 24 isolates (12.6%) were collected from elderly patients (≥65 years) and 61 isolates (31.9%) were collected from paediatric patients (aged ≤12 years).

Summary MIC and susceptibility data for all 191 *H. influenzae* are shown in Tables 7–9. MIC distribution data are given in Table S2.

**Table 7.** Summary MIC and susceptibility data for *H. influenzae* (*n* = 191) from Vietnam using CLSI breakpoints

Antimicrobial	MIC (mg/L)			CLSI susceptibility		
	Range	50%	90%	%S	%I	%R
Amoxicillin	0.25 to >128	8	>128	—	—	—
Ampicillin	0.12 to >128	8	>128	7.9	7.9	84.3
Amoxicillin/clavulanic acid (2:1)	0.25–16	4	8	32.5	50.3	17.3
Cefaclor	≤0.25 to >32	>32	>32	21.5	12.0	66.5
Cefdinir	≤0.06 to >4	4	>4	18.8	—	—
Cefixime	≤0.008 to >4	2	4	27.7	—	—
Cefotaxime	≤0.002–2	0.5	1	100	—	—
Cefpodoxime	≤0.015 to >4	4	4	49.2	—	—
Ceftibuten	≤0.008 to >4	>4	>4	13.1	—	—
Ceftriaxone	≤0.001–0.5	0.25	0.25	100	—	—
Cefuroxime	≤0.03 to >16	4	>16	62.8	11.0	26.2
Azithromycin	≤0.12 to >8	1	8	89.5	—	—
Clarithromycin	≤0.25 to >32	8	16	82.2	9.4	8.4
Tetracycline	≤0.12 to >32	0.25	16	83.2	1.6	15.2
Levofloxacin	≤0.004 to >8	0.015	8	89.0	—	—
Moxifloxacin	≤0.004 to >8	0.03	4	88.0	—	—
Trimethoprim/sulfamethoxazole	≤0.008 to >8	8	>8	20.4	0	79.6

—, not applicable; I, intermediate; R, resistant; S, susceptible.

**Table 8.** Summary MIC and susceptibility data for *H. influenzae* (*n* = 191) from Vietnam using EUCAST (dose-specific) breakpoints

	MIC (mg/L)			EUCAST susceptibility		
Antimicrobial	Range	50%	90%	%S	%I	%R
Amoxicillin (0.5 g × 3 oral)	0.25 to >128	8	>128	0	17.8	82.2
Amoxicillin (0.75–1 g × 3 oral)	0.25 to >128	8	>128	17.8	—	82.2
Ampicillin	0.12 to >128	8	>128	7.9	—	92.1
Amoxicillin/clavulanic acid (0.5 g/0.125 g × 3 oral)	0.12–8	4	4	0	45.5	54.5
Amoxicillin/clavulanic acid (0.875 g/0.125 g × 3 oral)	0.12–8	4	4	45.5	—	54.5
Cefaclor	≤0.25 to >32	>32	>32	—	—	—
Cefdinir	≤0.06 to >4	4	>4	—	—	—
Cefixime	≤0.008 to >4	2	4	9.4	—	90.6
Cefotaxime	≤0.002–2	0.5	1	15.7	—	84.3
Cefpodoxime	≤0.015 to >4	4	4	9.9	—	90.1
Ceftibuten	≤0.008 to >4	>4	>4	10.5	—	89.5
Ceftriaxone	≤0.001–0.5	0.25	0.25	44.5	—	55.5
Cefuroxime	≤0.03 to >16	4	>16	0	18.8	81.2
Azithromycin	≤0.12 to >8	1	8	—	—	—
Clarithromycin	≤0.25 to >32	8	16	—	—	—
Tetracycline	≤0.12 to >32	0.25	16	83.2	—	16.8
Levofloxacin	≤0.004 to >8	0.015	8	62.8	—	73.2
Moxifloxacin	≤0.004 to >8	0.03	4	69.6	—	30.4
Trimethoprim/sulfamethoxazole (0.16 g/0.8 g × 2 oral or IV)	≤0.008 to >8	8	>8	20.4	0	79.6
Trimethoprim/sulfamethoxazole (0.24 g/1.2 g × 2 oral or IV)	≤0.008 to >8	8	>8	20.4	—	79.6

—, not applicable; I, susceptible, increased exposure; R, resistant; S, susceptible.

**Table 9.** Summary MIC and susceptibility data for *H. influenzae* (*n* = 191) from Vietnam using PK/PD breakpoints

Antimicrobial	MIC (mg/L)			PK/PD susceptibility
	Range	50%	90%	%S
Amoxicillin (1.5 g/day)	0.25 to >128	8	>128	17.8
Amoxicillin (4 g/day)	0.25 to >128	8	>128	49.7
Amoxicillin/clavulanic acid (1.75 g/0.25 g/day adults; 45 mg/6.4 mg/kg/day children)	0.25–16	4	8	32.5
Amoxicillin/clavulanic acid (4 g/0.25 g/day adults; 90 mg/6.4 mg/kg/day children)	0.25–16	4	8	82.7
Ampicillin	0.12 to >128	8	>128	—
Cefaclor	≤0.25 to >32	>32	>32	0.5
Cefdinir	≤0.06 to >4	4	>4	6.8
Cefixime	≤0.008 to >4	2	4	27.7
Cefotaxime	≤0.002–2	0.5	1	—
Cefpodoxime	≤0.015 to >4	4	4	12.0
Ceftibuten	≤0.008 to >4	>4	>4	—
Ceftriaxone	≤0.001–0.5	0.25	0.25	100
Cefuroxime	≤0.03 to >16	4	>16	18.8
Azithromycin	≤0.12 to >8	1	8	5.8
Clarithromycin	≤0.25 to >32	8	16	3.7
Tetracycline	≤0.12 to >32	0.25	16	—
Levofloxacin	≤0.004 to >8	0.015	8	89.0
Moxifloxacin	≤0.004 to >8	0.03	4	88.0
Trimethoprim/sulfamethoxazole	≤0.008 to >8	8	>8	20.4

—, not applicable; PK/PD, pharmacokinetic/pharmacodynamic; S, susceptible.

### *H. influenzae* susceptibility

Among the 191 *H. influenzae* isolates collected, 59.7% were β-lactamase-negative (*n* = 114), of which 87.7% (*n* = 100) and 74.6% (*n* = 85) were β-lactamase-negative ampicillin-resistant (BLNAR) following EUCAST and CLSI criteria, respectively.

Overall high resistance to amoxicillin was 82.2% using EUCAST breakpoints (no CLSI breakpoints). Susceptibility was 49.7% by PK/PD using the higher dose (4 g/day). Susceptibility to amoxicillin/clavulanic acid (2:1) was 32.5% and 17.3% were resistant by CLSI. By EUCAST criteria, 45.5% were susceptible to high-dose amoxicillin/clavulanic acid (2 mg/L) (0.875 g/0.125 g × 3 oral). The low-dose [0.5 g every 8 h or 0.875 g every 12 h oral (amoxicillin component), CLSI] amoxicillin/clavulanic acid (2:1) resulted in a relatively large proportion of intermediate isolates (50.3%). However, PK/PD higher-dose amoxicillin/clavulanic acid (4 g/0.25 g/day) increased susceptibility to 82.7%. Resistance to ampicillin was 84.3% (CLSI) and 92.1% (EUCAST).

**Figure 4.**
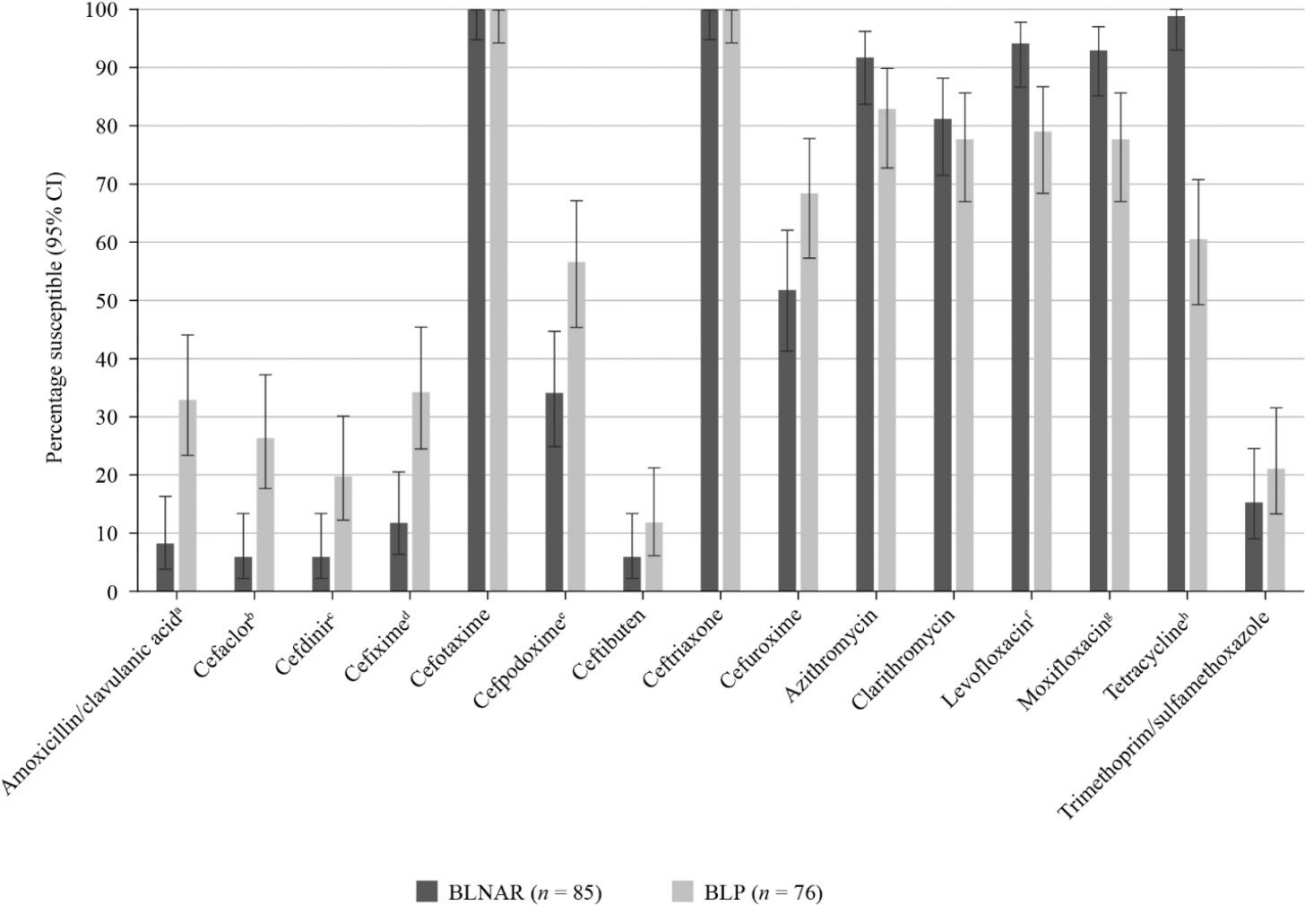
Comparison of antibiotic susceptibility rates of BLNAR and BLP ampicillin-resistant *H. influenzae* isolates from Vietnam collected in 2020–21 (CLSI breakpoints). Statistically significant decrease in susceptibility in BLNAR: ^a^*P* = 0.0019, ^b^*P* = 0.0004, ^c^*P* = 0.0089, ^d^*P* = 0.0011, ^e^*P* = 0.0046. Statistically significant increase in susceptibility in BLNAR: ^f^*P* = 0.0049, ^g^*P* = 0.0067, ^h^*P* < 0.0001. BLNAR, β-lactamase-negative ampicillin-resistant; BLP, β-lactamase-positive; CI, confidence interval.

**Figure 5.**
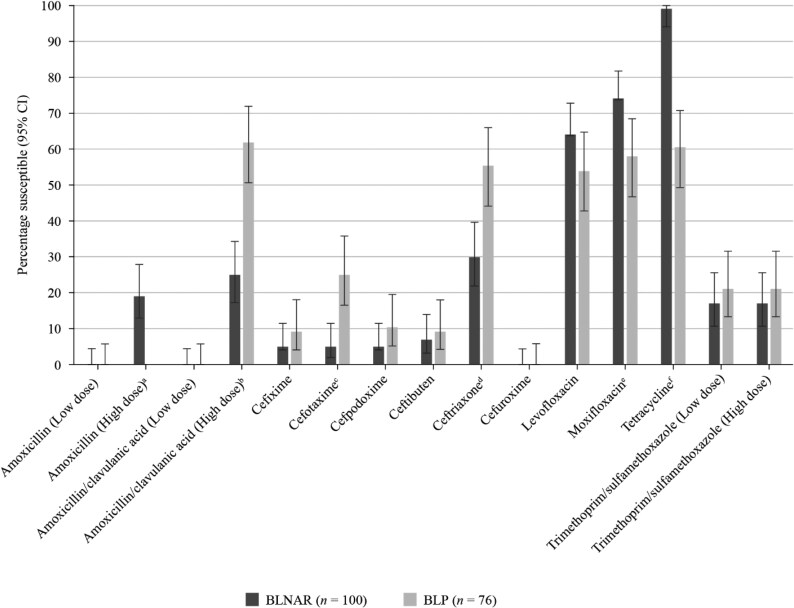
Comparison of antibiotic susceptibility rates of BLNAR and BLP ampicillin-resistant *H. influenzae* isolates from Vietnam collected in 2020–21 (EUCAST breakpoints). Statistically significant decrease in susceptibility in BLNAR: ^a^*P* < 0.0001, ^b^*P* < 0.0001, ^c^*P* = 0.0002, ^d^*P* = 0.0011. Statistically significant increase in susceptibility in BLNAR: ^e^*P* = 0.0349, ^f^*P* < 0.0001. BLNAR, β-lactamase-negative ampicillin-resistant; BLP, β-lactamase-positive; CI, confidence interval.

The cause of ampicillin resistance was evenly split between the 85 BLNAR and 76 β-lactamase-positive (BLP) isolates for both CLSI (52.8% versus 47.2%) and EUCAST (56.8% versus 43.2%). Amoxicillin/clavulanic acid was significantly more active against BLP than BLNAR by CLSI (32.9% versus 8.2%, *P* = 0.0019) and high-dose EUCAST breakpoints (61.8% versus 25.0%, *P* < 0.0001) (Figures 4 and 5). According to CLSI breakpoints, cefotaxime and ceftriaxone were fully active against BLP or BLNAR isolates. EUCAST breakpoints indicated significantly better activity against BLP than BLNAR for both of these third-generation cephalosporins. BLP susceptibility to cefotaxime and ceftriaxone was 25.0% and 55.3% by EUCAST, respectively (Figure 5). In most cases, β-lactam antimicrobial activity was greater against BLP isolates than BLNAR isolates. Significant differences in fluoroquinolone and tetracycline susceptibility also occurred between BLP and BLNAR isolates, but these tended to show better activity against BLNAR than BLP isolates (Figures 4 and 5).

Susceptibility of 100% was observed for *H. influenzae* against cefotaxime and ceftriaxone using CLSI breakpoints. However, when using EUCAST breakpoints, 15.7% and 44.5% of the isolates were susceptible, respectively. A large breakpoint-specific difference was also seen for cefuroxime (oral) with 62.8% susceptible isolates by CLSI, but none were susceptible by EUCAST and 18.8% were susceptible by PK/PD breakpoints. For cefpodoxime, the susceptibility rate was 49.2% (CLSI), 12.0% (PK/PD) and 9.9% (EUCAST). The other cephalosporins tested displayed poor activity with resistance rates of 66.5%–90.6% according to CLSI and EUCAST criteria.

**Figure 6.**
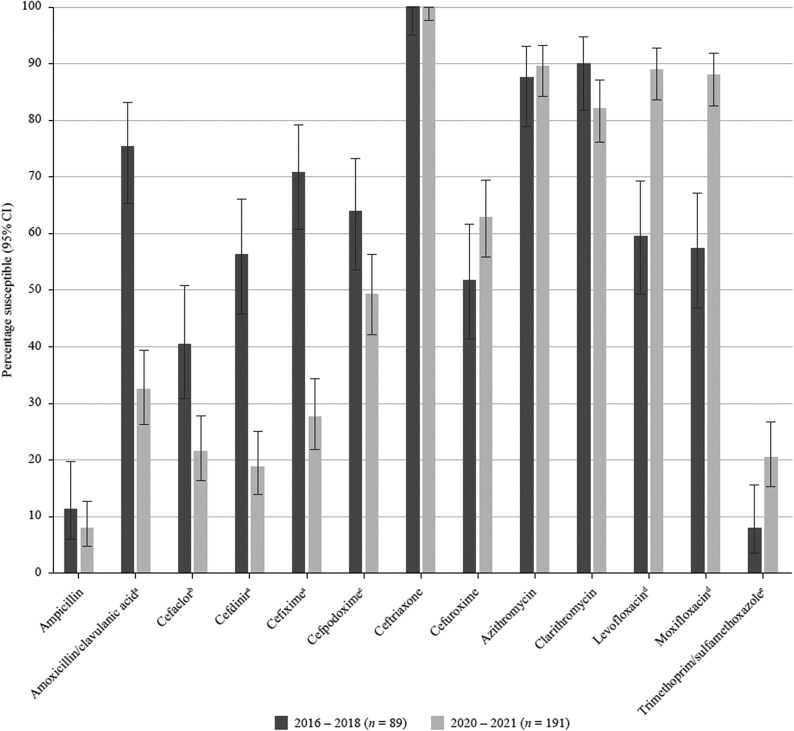
Comparison of antibiotic susceptibility rates of *H. influenzae* isolates from Vietnam collected in 2016–18 with isolates collected in 2020–21 (CLSI breakpoints). Statistically significant decrease in susceptibility: ^a^*P* < 0.0001, ^b^*P* = 0.001, ^c^*P* = 0.021. Statistically significant increase in susceptibility: ^d^*P* < 0.0001, ^e^*P* = 0.009.

**Figure 7.**
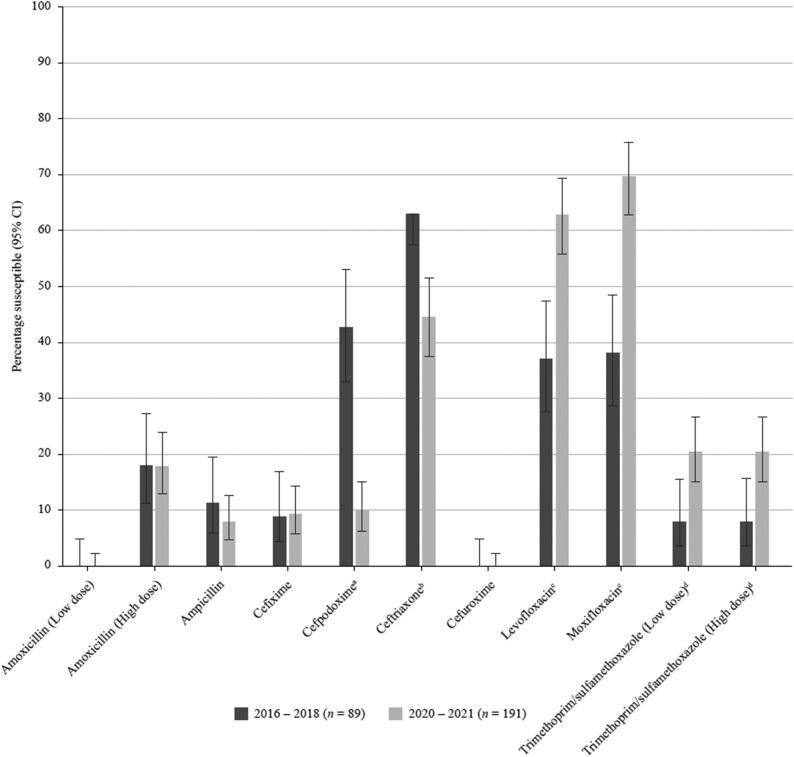
Comparison of antibiotic susceptibility rates of *H. influenzae* isolates from Vietnam collected in 2016–18 with isolates collected in 2020–21 (EUCAST breakpoints). Statistically significant decrease in susceptibility: ^a^*P* < 0.0001, ^b^*P* = 0.005. Statistically significant increase in susceptibility: ^c^*P* < 0.0001, ^d^*P* = 0.009. CI, confidence interval.

Levofloxacin and moxifloxacin were active against 89.0% and 88.0% of the isolates, respectively, using CLSI or PK/PD breakpoints, but susceptibility was reduced to 62.8% and 69.6%, respectively, when applying EUCAST criteria. Isolate susceptibility to the macrolides was 82.2% (clarithromycin) and 89.5% (azithromycin) using CLSI criteria, but 3.7% and 5.8% by PK/PD, respectively; no breakpoints were published by EUCAST. Overall, 83.2% of the *H. influenzae* isolates were tetracycline-susceptible according to CLSI and EUCAST criteria. Susceptibility to trimethoprim/sulfamethoxazole was 20.4% by all breakpoints (Tables 7–9, Figures 6 and 7).

## Discussion

The increasing prevalence of AMR among the major pathogens responsible for CA-RTIs is a serious global problem that complicates the management of these infections. The present study was conducted with the primary objective of generating local resistance patterns among *S. pneumoniae* and *H. influenzae* collected in Vietnam during 2020–21 from patients with CA-RTIs.

While the study’s geographic scope is limited, with samples collected from only two sites in one region, the predominance of isolates from presumably unrelated community-acquired infections supports the assumption that the findings may be representative of the broader antibiotic resistance patterns in Vietnam, at least within the Ho Chi Minh City region. Another limitation of this study is the lack of disease severity data. Sample collection occurred during the COVID-19 outbreak, which may have had an influence on the prevalence of antibiotic resistance in Vietnam. Additionally, the detection of the mechanism of BLNAR is limited; therefore, it is not known how *H. influenzae* is resistant to amoxicillin/clavulanic acid and cephalosporins.

Vietnam data from this current SOAR study highlight important features with respect to contemporary surveillance in Ho Chi Minh City. As defined by CLSI oral and EUCAST low-dose penicillin breakpoints, only 6.3% of the *S. pneumoniae* isolates were susceptible, although this represents a small but significant increase (*P* = 0.028) compared with the previous SOAR (2016–18) surveillance study in Vietnam which included data from the same hospitals as in the current study.^8^ When applying EUCAST high-dose or CLSI IV breakpoints, the rate of penicillin susceptibility increased to 69.5%, which was essentially the same rate as in the previous SOAR (2016–18) study.^8^ This does, however, indicate a substantial drop in pneumococcal penicillin susceptibility compared with the SOAR surveillance study in 2009–11 where 86.9% susceptibility (CLSI IV) was observed.^13^ Although the comparability of the data from two sites in the current study with the study in 2009–11 performed in 11 different centres is limited, the increase in penicillin resistance among *S. pneumoniae* isolates in Vietnam is concerning, and stringent national antibiotic surveillance (using accredited laboratories in Vietnam) is warranted.

By far the highest pneumococcal susceptibility was seen to the fluoroquinolones (>95% by CLSI, EUCAST or PK/PD breakpoints). Fluoroquinolone susceptibility of *H. influenzae* was 83%–89% by CLSI or PK/PD and 62%–70% by EUCAST criteria. Interestingly, according to CLSI and EUCAST, there was a significant increase in levofloxacin and moxifloxacin susceptibility (*P* < 0.0001) compared with the 2016–18 data where the rate was <60% (CLSI) or <40% (EUCAST). This improved activity may relate to reduced use of fluoroquinolones. Although this class of antibacterials may be an appropriate therapy for CA-RTIs, fluoroquinolones must be prescribed with caution to avoid possible severe side effects and the redevelopment of resistance in the future.

According to EUCAST criteria, amoxicillin/clavulanic acid (fixed 2 mg/L) (high-dose) susceptibility in pneumococci remained stable between the 2016–18 study and the present surveillance study at 26.1% and 25.7%, respectively. Similarly, when applying CLSI breakpoints, *S. pneumoniae* susceptibility to amoxicillin/clavulanic acid (2:1) was only moderately reduced from 59.6% in 2016–18 to 56.3% in the present study. Pneumococcal susceptibility to PK/PD high-dose amoxicillin (4 g/day) or amoxicillin/clavulanic acid (4 g/0.25 g/day) was also relatively stable at 72.1% in 2016–18 and 78.5% in the present study. Nevertheless, this represents an important drop compared with data from SOAR in 2009–11, where 96.9% of pneumococci were categorized as susceptible by PK/PD high-dose breakpoints.^13^

In parallel, the rate of amoxicillin/clavulanic acid (2:1) intermediate and resistant *H. influenzae* has increased since the SOAR surveillance in 2016–18; the present study shows a significant drop (*P* < 0.0001) in susceptibility from 75.3% to 32.5%. It seems that the susceptible portion is gradually replaced by a growing intermediate population that will eventually develop full resistance in the future. However, this intermediate population would be susceptible to PK/PD higher-dose amoxicillin/clavulanic acid (4 g/0.25 g/day). The rate of amoxicillin/clavulanic acid (2:1) intermediate *H. influenzae* in the current study was 50.3%. Here we note that the CLSI amoxicillin/clavulanic acid (2:1) breakpoints were changed in 2021 by lowering the susceptible breakpoint from MIC ≤4 to ≤2 mg/L, which would reduce the susceptible fraction. However, the resistant breakpoint did not change, and a significant increase in the resistant portion from 4.5% to 17.3% (*P* = 0.006) is observed between the previous and the current SOAR study. The increasing resistance to this fixed-dose combination necessitates exploration of the role of higher ratios of amoxicillin/clavulanic acid that could counteract clinical failure due to resistance. In Vietnam, different ratios of amoxicillin/clavulanic acid such as 4:1, 7:1 and 8:1 are available. Currently, in some other countries, ratios of amoxicillin/clavulanic acid up to 14:1 are also in use. Higher doses of amoxicillin and amoxicillin/clavulanic acid might help to mitigate further development of resistance in Vietnam. This would require the evaluation of additional breakpoints for these alternative ratios and doses. Also, the combination of amoxicillin/clavulanic acid with an additional antimicrobial could improve clinical outcomes and prevent resistance development.

Other β-lactams (cefaclor, cefdinir and cefuroxime) were less active regardless of the breakpoints used.

Compared with the SOAR study in 2016–18, a significant increase in pneumococcal susceptibility to cefaclor (*P* = 0.011), cefdinir (*P* = 0.029) and cefpodoxime (*P* = 0.0003) was observed, though at a low level (<20%), using CLSI criteria. These trends were also seen when applying EUCAST breakpoints, with a significant increase of susceptibility to cefpodoxime (*P* = 0.0002) and cefuroxime (*P* = 0.03). In contrast, a significant decrease in pneumococcal ceftriaxone susceptibility (*P* = 0.05) from 62.1% in 2016–18 to 50.7% in 2020–21 was seen according to CLSI.

Contrary to the trends seen for pneumococci, the comparison with the SOAR study from 2016–18 shows that the susceptibility of *H. influenzae* to most cephalosporins has decreased. According to CLSI criteria, a significant decrease was seen for cefaclor (40.4% versus 21.5%, *P* = 0.001), cefdinir (56.2% versus 18.8%, *P* < 0.0001), cefixime (70.8% versus 27.7%, *P* < 0.0001) and cefpodoxime (64.0% versus 49.2%, *P* = 0.021). When applying EUCAST criteria, a significant decrease in susceptibility is confirmed for cefpodoxime (42.7% versus 9.9%, *P* < 0.0001) and ceftriaxone (62.9% versus 44.5%, *P* = 0.005). In contrast to the EUCAST susceptibility rates for ceftriaxone (44.5%) and cefotaxime (15.7%), susceptibility according to CLSI criteria was 100% for both compounds. This is a good example of where breakpoint harmonization between CLSI and EUCAST is required.

As in previous years, macrolides and trimethoprim/sulfamethoxazole displayed very poor activity against *S. pneumoniae*. In contrast, macrolide susceptibility of *H. influenzae* was 80%–90% (CLSI) and comparable to the rates observed in SOAR 2016–18.^8^ For clinical practice, this suggests macrolides may not be a valuable option for monotherapy if there is uncertainty over the cause of infection due to *S. pneumoniae* or *H. influenzae*.

Among the isolates of *H. influenzae*, the proportion of β-lactamase-negative strains (59.7%) was higher than in the 2016–18 SOAR study (32.6%) but comparable to the rate from the SOAR study of 2009–11.^13^ However, the rate of BLNAR greatly increased from a total of 15.7% in the previous study to 44.5% of *H. influenzae* in the present study (CLSI breakpoints). These BLNAR are also less susceptible to β-lactam agents than BLP isolates but more susceptible to fluoroquinolones and tetracyclines.

Unlike the previous SOAR study (2016–18), in which a relatively large proportion of isolates were from paediatric patients (72.0% of *S. pneumoniae* and 75.3% of *H. influenzae*), the current study shows more isolates coming from adolescent/adult/elderly patients (54.9% of *S. pneumoniae* and 68.1% of *H. influenzae*).

The SOAR study initiative serves as a pivotal tool in understanding the evolving landscape of AMR among the key respiratory pathogens *S. pneumoniae* and *H. influenzae* in Vietnam. The comprehensive surveillance efforts presented in this study shed light on the complex patterns of susceptibility and resistance across different strain populations and provide nuanced insights into the efficacy of various antibiotics, highlighting the importance of prudent antibiotic use, tailored treatment approaches and the critical role of vaccination as a method to prevent infections. The incorporation of multiple interpretative breakpoints, including those recommended by CLSI, EUCAST and PK/PD interpretative criteria, adds to our understanding of susceptibility dynamics. As AMR continues to pose significant challenges to global health, the findings presented in this survey underscore the critical need for ongoing surveillance, collaborative efforts and informed antibiotic stewardship to address the escalating threat of AMR and to ensure effective management of respiratory tract infections.

The SOAR study continues to evolve; in the present surveillance, both sites were from Ho Chi Minh City and therefore may not capture the antibiotic resistance trends in other regions within Vietnam. Future SOAR surveillance will need to include additional sites and an increased number of isolates with clinical presentation. Future data from Vietnam will further assist in understanding the longitudinal implications related to AMR in this country. Addition of clinical and patient outcome data would provide considerable added value to pathogen-based surveillance, as would continuous monitoring of local antibiotic use and bacterial resistance patterns.

## Supplementary Material

dkaf290_Supplementary_Data

## Supplementary data

Tables S1 and S2 are available as Supplementary data at *JAC* Online.
