# Supplementary material for: Results from the Survey of Antibiotic Resistance (SOAR) 2020–21 in Vietnam: data based on CLSI, EUCAST (dose-specific) and pharmacokinetic/pharmacodynamic (PK/PD) breakpoints
Source: J Antimicrob Chemother. 2025 Nov 24;80(Suppl 3):iii114–28. doi: 10.1093/jac/dkaf290 (PMC12641131; doi:10.1093/jac/dkaf290)
Supplement: dkaf290_Supplementary_Data [file dkaf290_supplementary_data.docx]

**Results from the Survey of Antibiotic Resistance (SOAR) 2020 – 21 in Vietnam: data based on CLSI, EUCAST (dose-specific) and pharmacokinetic/pharmacodynamic (PK/PD) breakpoints**

**Authors:** Didem TORUMKUNEY^1^, Pham Hung VAN^2^, Le Quoc THINH^3^, Stephen HAWSER^4^, Subhashri KUNDU^5^, Ngoc TRUONG HA LAN^6^, Anand MANOHARAN^7*^

**Affiliations:** ^1^GSK, London, UK; ^2^Vietnam Research and Development Institute of Clinical Microbiology (VCM), Ho Chi Minh City, Vietnam; ^3^Pediatric 1 Hospital, Ho Chi Minh City, Vietnam; ^4^IHMA Europe Sàrl, Monthey, Switzerland; ^5^GSK, Singapore; ^6^GSK, Ho Chi Minh City, Vietnam; ^7^Infectious Diseases Medical & Scientific Affairs, GSK, Mumbai, India

*Corresponding author. E-mail: [anand.x.manoharan@gsk.com](mailto:anand.x.manoharan@gsk.com)

**Running title:** Survey of Antibiotic Resistance (SOAR) in Vietnam in 2020 – 21

**Supplementary Table 1.** MIC distribution for *S. pneumoniae* isolates (*n* = 144) from Vietnam

|  |  | Number of isolates at MIC (mg/L) | | | | | | | | | | | | | | | | | | | | |
| --- | --- | --- | --- | --- | --- | --- | --- | --- | --- | --- | --- | --- | --- | --- | --- | --- | --- | --- | --- | --- | --- | --- |
| Antimicrobial |  | ≤0.008 | ≤0.015 | 0.015 | ≤0.03 | 0.03 | ≤0.06 | 0.06 | ≤0.12 | 0.12 | ≤0.25 | 0.25 | ≤0.5 | 0.5 | 1 | 2 | 4 | >4 | 8 | >8 | 16 | >16 |
| AMX | N | – | – | 4 | – | 3 | – | 1 | – | 4 | – | 7 | – | 7 | 16 | 40 | 31 | – | 30 | 1 | – | – |
|  | Cum. % | – | – | 2.8 | – | 4.9 | – | 5.6 | – | 8.4 | – | 13.3 | – | 18.2 | 29.3 | 57.1 | 78.6 | – | 99.4 | 100 | – | – |
|  | % | – | – | 2.8 | – | 2.1 | – | 0.7 | – | 2.8 | – | 4.9 | – | 4.9 | 11.1 | 27.8 | 21.5 | – | 20.8 | 0.7 | – | – |
| AMC (2:1) | N | 2 | – | 2 | – | 4 | – | 1 | – | 3 | – | 7 | – | 7 | 19 | 36 | 32 | – | 29 | 2 | – | – |
|  | Cum. % | 1.4 | – | 2.8 | – | 5.6 | – | 6.3 | – | 8.4 | – | 13.3 | – | 18.2 | 31.4 | 56.4 | 78.6 | – | 98.7 | 100 | – | – |
|  | % | 1.4 | – | 1.4 | – | 2.8 | – | 0.7 | – | 2.1 | – | 4.9 | – | 4.9 | 13.2 | 25.0 | 22.2 | – | 20.1 | 1.4 | – | – |
| AMC | N | 5 | – | – | – | 3 | – | 2 | – | 2 | – | 4 | – | 8 | 13 | 40 | 27 | – | 35 | 5 | – | – |
| [2 mg/L] | Cum. % | 3.5 | – | – | – | 4.6 | – | 7.0 | – | 8.4 | – | 11.2 | – | 16.8 | 25.8 | 53.6 | 72.4 | – | 96.7 | 100 | – | – |
|  | % | 3.5 | – | – | – | 2.1 | – | 1.4 | – | 1.4 | – | 2.8 | – | 5.6 | 9.0 | 27.8 | 18.8 | – | 24.3 | 3.5 | – | – |
| AZM | N | – | 1 | – | – | 3 | – | 4 | – | – | – | 1 | – | 1 | 1 | 2 | 4 | – | 2 | – | 3 | 122 |
|  | Cum. % | – | 0.7 | – | – | 2.8 | – | 5.6 | – | – | – | 6.3 | – | 7 | 7.7 | 9.1 | 11.9 | – | 13.3 | – | 15.4 | 100 |
|  | % | – | 0.7 | – | – | 2.1 | – | 2.8 | – | – | – | 0.7 | – | 0.7 | 0.7 | 1.4 | 2.8 | – | 1.4 | – | 2.1 | 84.7 |
| CEC | N | – | – | – | – | 1 | – | – | – | – | – | 2 | – | 6 | 6 | 6 | 3 | 120 | – | – | – | – |
|  | Cum. % | – | – | – | – | 0.7 | – | – | – | – | – | 2.1 | – | 6.3 | 10.5 | 14.7 | 16.8 | 100 | – | – | – | – |
|  | % | – | – | – | – | 0.7 | – | – | – | – | – | 1.4 | – | 4.2 | 4.2 | 4.2 | 2.1 | 83.3 | – | – | – | – |
| CDR | N | – | 2 | – | – | 1 | – | 6 | – | 4 | – | 5 | – | 8 | 3 | 12 | 35 | – | 50 | 18 | – | – |
|  | Cum. % | – | 1.4 | – | – | 2.1 | – | 6.3 | – | 9.1 | – | 12.6 | – | 18.2 | 20.3 | 28.6 | 52.9 | – | 87.6 | 100 | – | – |
|  | % | – | 1.4 | – | – | 0.7 | – | 4.2 | – | 2.8 | – | 3.5 | – | 5.6 | 2.1 | 8.3 | 24.3 | – | 34.7 | 12.5 | – | – |
| CFM | N | – | – | – | – | – | – | – | – | – | 11 | – | – | 2 | 7 | 6 | 7 | – | 13 | – | 39 | 59 |
|  | Cum. % | – | – | – | – | – | – | – | – | – | 7.6 | – | – | 9 | 13.9 | 18.1 | 23.0 | – | 32.0 | – | 59.1 | 100 |
|  | % | – | – | – | – | – | – | – | – | – | 7.6 | – | – | 1.4 | 4.9 | 4.2 | 4.9 | – | 9.0 | – | 27.1 | 41.0 |
| CTX | N | 3 | – | 3 | – | 2 | – | 1 | – | 10 | – | 9 | – | 15 | 46 | 45 | 6 | 4 | – | – | – | – |
|  | Cum. % | 2.1 | – | 4.2 | – | 5.6 | – | 6.3 | – | 13.2 | – | 19.5 | – | 29.9 | 61.8 | 93.1 | 97.3 | 100 | – | – | – | – |
|  | % | 2.1 | – | 2.1 | – | 1.4 | – | 0.7 | – | 6.9 | – | 6.3 | – | 10.4 | 31.9 | 31.3 | 4.2 | 2.8 | – | – | – | – |
| CPD | N | – | 1 | – | – | 6 | – | 2 | – | 7 | – | 4 | – | 8 | 15 | 39 | 46 | 16 | – | – | – | – |
|  | Cum. % | – | 0.7 | – | – | 4.9 | – | 6.3 | – | 11.2 | – | 14.0 | – | 19.6 | 30.0 | 57.1 | 89.0 | 100 | – | – | – | – |
|  | % | – | 0.7 | – | – | 4.2 | – | 1.4 | – | 4.9 | – | 2.8 | – | 5.6 | 10.4 | 27.1 | 31.9 | 11.1 | – | – | – | – |
| CTB | N | – | – | – | – | – | – | – | – | – | – | – | 2 | – | – | – | 6 | – | 2 | – | 9 | 125 |
|  | Cum. % | – | – | – | – | – | – | – | – | – | – | – | 1.4 | – | – | – | 5.6 | – | 7 | – | 13.3 | 100 |
|  | % | – | – | – | – | – | – | – | – | – | – | – | 1.4 | – | – | – | 4.2 | – | 1.4 | – | 6.3 | 86.8 |
| CRO | N | – | – | 1 | – | 6 | – | 2 | – | 3 | – | 8 | – | 15 | 38 | 59 | 10 | 2 | – | – | – | – |
|  | Cum. % | – | – | 0.7 | – | 4.9 | – | 6.3 | – | 8.4 | – | 14 | – | 24.4 | 50.8 | 91.8 | 98.7 | 100 | – | – | – | – |
|  | % | – | – | 0.7 | – | 4.2 | – | 1.4 | – | 2.1 | – | 5.6 | – | 10.4 | 26.4 | 41.0 | 6.9 | 1.4 | – | – | – | – |
| CXM | N | 1 | – | 3 | – | 1 | – | 2 | – | 8 | – | 1 | – | 2 | 5 | 16 | 44 | – | 40 | 21 | – | – |
|  | Cum. % | 0.7 | – | 2.8 | – | 3.5 | – | 4.9 | – | 10.5 | – | 11.2 | – | 12.6 | 16.1 | 27.2 | 57.8 | – | 85.6 | 100 | – | – |
|  | % | 0.7 | – | 2.1 | – | 0.7 | – | 1.4 | – | 5.6 | – | 0.7 | – | 1.4 | 3.5 | 11.1 | 30.6 | – | 27.8 | 14.6 | – | – |
| CLR | N | – | 4 | – | – | 3 | – | 1 | – | 1 | – | 2 | – | 1 | 4 | 4 | 2 | – | 2 | – | 2 | 118 |
|  | Cum. % | – | 2.8 | – | – | 4.9 | – | 5.6 | – | 6.3 | – | 7.7 | – | 8.4 | 11.2 | 14 | 15.4 | – | 16.8 | – | 18.2 | 100 |
|  | % | – | 2.8 | – | – | 2.1 | – | 0.7 | – | 0.7 | – | 1.4 | – | 0.7 | 2.8 | 2.8 | 1.4 | – | 1.4 | – | 1.4 | 81.9 |
| DOX | N | 2 | – | – | – | 2 | – | 6 | – | 9 | – | – | – | 2 | 10 | 18 | 65 | 30 | – | – | – | – |
|  | Cum. % | 1.4 | – | – | – | 2.8 | – | 7 | – | 13.3 | – | – | – | 14.7 | 21.6 | 34.1 | 79.2 | 100 | – | – | – | – |
|  | % | 1.4 | – | – | – | 1.4 | – | 4.2 | – | 6.3 | – | – | – | 1.4 | 6.9 | 12.5 | 45.1 | 20.8 | – | – | – | – |
| ERY | N | – | 2 | – | – | 5 | – | – | – | 1 | – | – | – | 1 | 2 | 5 | 3 | – | 1 | – | 1 | 123 |
|  | Cum. % | – | 1.4 | – | – | 4.9 | – | – | – | 5.6 | – | – | – | 6.3 | 7.7 | 11.2 | 13.3 | – | 14 | – | 14.7 | 100 |
|  | % | – | 1.4 | – | – | 3.5 | – | – | – | 0.7 | – | – | – | 0.7 | 1.4 | 3.5 | 2.1 | – | 0.7 | – | 0.7 | 85.4 |
| LVX | N | – | – | – | – | – | – | – | 2 | – | – | – | – | 46 | 86 | 4 | – | – | 1 | 5 | – | – |
|  | Cum. % | – | – | – | – | – | – | – | 1.4 | – | – | – | – | 33.3 | 93.0 | 95.8 | – | – | 96.5 | 100 | – | – |
|  | % | – | – | – | – | – | – | – | 1.4 | – | – | – | – | 31.9 | 59.7 | 2.8 | – | – | 0.7 | 3.6 | – | – |
| MXF | N | – | – | – | 3 | – | – | 62 | – | 72 | – | – | – | – | – | 3 | 1 | 3 | – | – | – | – |
|  | Cum. % | – | – | – | 2.1 | – | – | 45.2 | – | 95.2 | – | – | – | – | – | 97.3 | 98 | 100 | – | – | – | – |
|  | % | – | – | – | 2.1 | – | – | 43.1 | – | 50.0 | – | – | – | – | – | 2.1 | 0.7 | 2.1 | – | – | – | – |
| PEN | N | – | – | 4 | – | 3 | – | 2 | – | 6 | – | 7 | – | 5 | 20 | 53 | 41 | – | 3 | – | – | – |
|  | Cum. % | – | – | 2.8 | – | 4.9 | – | 6.3 | – | 10.5 | – | 15.4 | – | 18.9 | 32.8 | 69.6 | 98.1 | – | 100 | – | – | – |
|  | % | – | – | 2.8 | – | 2.1 | – | 1.4 | – | 4.2 | – | 4.9 | – | 3.5 | 13.9 | 36.8 | 28.5 | – | 2.1 | – | – | – |
| TET | N | – | – | – | 1 | – | – | 2 | – | 7 | – | 8 | – | 1 | 1 | 1 | 3 | 120 | – | – | – | – |
|  | Cum. % | – | – | – | 0.7 | – | – | 2.1 | – | 7 | – | 12.6 | – | 13.3 | 14.0 | 14.7 | 16.8 | 100 | – | – | – | – |
|  | % | – | – | – | 0.7 | – | – | 1.4 | – | 4.9 | – | 5.6 | – | 0.7 | 0.7 | 0.7 | 2.1 | 83.3 | – | – | – | – |
| SXT | N | – | – | – | – | – | 9 | – | – | 6 | – | 4 | – | 3 | 2 | 9 | 61 | – | 30 | 20 | – | – |
|  | Cum. % | – | – | – | – | – | 6.3 | – | – | 10.5 | – | 13.3 | – | 15.4 | 16.8 | 23.1 | 65.5 | – | 86.3 | 100 | – | – |
|  | % | – | – | – | – | – | 6.3 | – | – | 4.2 | – | 2.8 | – | 2.1 | 1.4 | 6.3 | 42.4 | – | 20.8 | 13.9 | – | – |

–, not applicable; AMC, amoxicillin/clavulanic acid; AMX, amoxicillin; AZM, azithromycin; CDR, cefdinir; CEC, cefaclor; CFM, cefixime; CLR, clarithromycin; CPD, cefpodoxime; CRO, ceftriaxone; CTB, ceftibuten; CTX, cefotaxime; Cum., cumulative; CXM, cefuroxime; DOX, doxycycline; ERY, erythromycin; LVX, levofloxacin; MXF, moxifloxacin; PEN, penicillin; SXT, trimethoprim/sulfamethoxazole; TET, tetracycline.

Bold vertical bars in table correspond to the CLSI-susceptible breakpoints.

**Supplementary Table 2.** MIC distribution for *H. influenzae* isolates (*n* = 191) from Vietnam

|  | |  | | Number of isolates at MIC (mg/L) | | | | | | | | | | | | | | | | | | | | | | | | | | | | | | | | | | | | | | | | |  |
| --- | --- | --- | --- | --- | --- | --- | --- | --- | --- | --- | --- | --- | --- | --- | --- | --- | --- | --- | --- | --- | --- | --- | --- | --- | --- | --- | --- | --- | --- | --- | --- | --- | --- | --- | --- | --- | --- | --- | --- | --- | --- | --- | --- | --- | --- |
| Antimicrobial | |  | | ≤0.001 | | ≤0.002 | | 0.002 | | ≤0.004 | | 0.004 | | ≤0.008 | | 0.008 | | ≤0.015 | | 0.015 | | ≤0.03 | | 0.03 | ≤0.06 | 0.06 | ≤0.12 | 0.12 | ≤0.25 | 0.25 | 0.5 | 1 | 2 | 4 | >4 | 8 | >8 | 16 | >16 | 32 | >32 | 64 | 128 | > 128 |  |
| AMX | N | | – | | – | | – | | – | | – | | – | | – | | – | | – | | – | | – | | – | – | – | – | – | 1 | 7 | 12 | 14 | 61 | – | 24 | – | 8 | – | 10 | – | 18 | 14 | 22 | |
|  | Cum. % | | – | | – | | – | | – | | – | | – | | – | | – | | – | | – | | – | | – | – | – | – | – | 0.5 | 4.2 | 10.5 | 17.8 | 49.7 | – | 62.3 | – | 66.5 | – | 71.7 | – | 81.1 | 88.4 | 100 | |
|  | % | | – | | – | | – | | – | | – | | – | | – | | – | | – | | – | | – | | – | – | – | – | – | 0.5 | 3.7 | 6.3 | 7.3 | 31.9 | – | 12.6 | – | 4.2 | – | 5.2 | – | 9.4 | 7.3 | 11.5 | |
| AMC (2:1) | N | | – | | – | | – | | – | | – | | – | | – | | – | | – | | – | | – | | – | – | – | – | – | 1 | 8 | 22 | 31 | 96 | – | 31 | – | 2 | – | – | – | – | – | – | |
|  | Cum. % | | – | | – | | – | | – | | – | | – | | – | | – | | – | | – | | – | | – | – | – | – | – | 0.5 | 4.7 | 16.2 | 32.4 | 82.7 | – | 98.9 | – | 100 | – | – | – | – | – | – | |
|  | % | | – | | – | | – | | – | | – | | – | | – | | – | | – | | – | | – | | – | – | – | – | – | 0.5 | 4.2 | 11.5 | 16.2 | 50.3 | – | 16.2 | – | 1.0 | – | – | – | – | – | – | |
| AMC | N | | – | | – | | – | | – | | – | | – | | – | | – | | – | | – | | – | | – | – | – | 1 | – | 9 | 14 | 22 | 41 | 90 | – | 14 | – | – | – | – | – | – | – | – | |
| [2 mg/L] | Cum. % | | – | | – | | – | | – | | – | | – | | – | | – | | – | | – | | – | | – | – | – | 0.5 | – | 5.2 | 12.5 | 24.0 | 45.5 | 92.6 | – | 100 | – | – | – | – | – | – | – | – | |
|  | % | | – | | – | | – | | – | | – | | – | | – | | – | | – | | – | | – | | – | – | – | 0.5 | – | 4.7 | 7.3 | 11.5 | 21.5 | 47.1 | – | 7.3 | – | – | – | – | – | – | – | – | |
| AMP | N | | – | | – | | – | | – | | – | | – | | – | | – | | – | | – | | – | | – | – | – | 1 | – | 4 | 1 | 9 | 15 | 35 | – | 49 | – | 8 | – | 7 | – | 13 | 23 | 26 | |
|  | Cum. % | | – | | – | | – | | – | | – | | – | | – | | – | | – | | – | | – | | – | – | – | 0.5 | – | 2.6 | 3.1 | 7.8 | 15.7 | 34 | – | 59.7 | – | 63.9 | – | 67.6 | – | 74.4 | 86.4 | 100 | |
|  | % | | – | | – | | – | | – | | – | | – | | – | | – | | – | | – | | – | | – | – | – | 0.5 | – | 2.1 | 0.5 | 4.7 | 7.9 | 18.3 | – | 25.7 | – | 4.2 | – | 3.7 | – | 6.8 | 12.0 | 13.6 | |
| AZM | N | | – | | – | | – | | – | | – | | – | | – | | – | | – | | – | | – | | – | – | 11 | – | – | 2 | 59 | 62 | 21 | 16 | – | 1 | 19 | – | – | – | – | – | – | – | |
|  | Cum. % | | – | | – | | – | | – | | – | | – | | – | | – | | – | | – | | – | | – | – | 5.8 | – | – | 6.8 | 37.7 | 70.2 | 81.2 | 89.6 | – | 90.1 | 100 | – | – | – | – | – | – | – | |
|  | % | | – | | – | | – | | – | | – | | – | | – | | – | | – | | – | | – | | – | – | 5.8 | – | – | 1.0 | 30.9 | 32.5 | 11.0 | 8.4 | – | 0.5 | 9.9 | – | – | – | – | – | – | – | |
| CEC | N | | – | | – | | – | | – | | – | | – | | – | | – | | – | | – | | – | | – | – | – | – | 1 | – | – | 1 | 7 | 7 | – | 25 | – | 23 | – | 17 | 110 | – | – | – | |
|  | Cum. % | | – | | – | | – | | – | | – | | – | | – | | – | | – | | – | | – | | – | – | – | – | 0.5 | – | – | 1 | 4.7 | 8.4 | – | 21.5 | – | 33.5 | – | 42.4 | 100 | – | – | – | |
|  | % | | – | | – | | – | | – | | – | | – | | – | | – | | – | | – | | – | | – | – | – | – | 0.5 | – | – | 0.5 | 3.7 | 3.7 | – | 13.1 | – | 12.0 | – | 8.9 | 57.6 | – | – | – | |
| CDR | N | | – | | – | | – | | – | | – | | – | | – | | – | | – | | – | | – | | 8 | – | – | – | – | 5 | 5 | 18 | 39 | 58 | 58 | – | – | – | – | – | – | – | – | – | |
|  | Cum. % | | – | | – | | – | | – | | – | | – | | – | | – | | – | | – | | – | | 4.2 | – | – | – | – | 6.8 | 9.4 | 18.8 | 39.2 | 69.6 | 100 | – | – | – | – | – | – | – | – | – | |
|  | % | | – | | – | | – | | – | | – | | – | | – | | – | | – | | – | | – | | 4.2 | – | – | – | – | 2.6 | 2.6 | 9.4 | 20.4 | 30.4 | 30.4 | – | – | – | – | – | – | – | – | – | |
| CFM | N | | – | | – | | – | | – | | – | | 7 | | – | | – | | 1 | | – | | 7 | | – | 3 | – | – | – | 2 | 2 | 31 | 51 | 72 | 15 | – | – | – | – | – | – | – | – | – | |
|  | Cum. % | | – | | – | | – | | – | | – | | 3.7 | | – | | – | | 4.2 | | – | | 7.9 | | – | 9.5 | – | – | – | 10.5 | 11.5 | 27.7 | 54.4 | 92.1 | 100 | – | – | – | – | – | – | – | – | – | |
|  | % | | – | | – | | – | | – | | – | | 3.7 | | – | | – | | 0.5 | | – | | 3.7 | | – | 1.6 | – | – | – | 1.0 | 1.0 | 16.2 | 26.7 | 37.7 | 7.9 | – | – | – | – | – | – | – | – | – | |
| CTX | N | | – | | 1 | | – | | – | | – | | – | | 1 | | – | | 7 | | – | | 1 | | – | 5 | – | 15 | – | 27 | 65 | 60 | 9 | – | – | – | – | – | – | – | – | – | – | – | |
|  | Cum. % | | – | | 0.5 | | – | | – | | – | | – | | 1.0 | | – | | 4.7 | | – | | 5.2 | | – | 7.8 | – | 15.7 | – | 29.8 | 63.8 | 95.2 | 100 | – | – | – | – | – | – | – | – | – | – | – | |
|  | % | | – | | 0.5 | | – | | – | | – | | – | | 0.5 | | – | | 3.7 | | – | | 0.5 | | – | 2.6 | – | 7.9 | – | 14.1 | 34.0 | 31.4 | 4.7 | – | – | – | – | – | – | – | – | – | – | – | |
| CPD | N | | – | | – | | – | | – | | – | | – | | – | | 7 | | – | | – | | 3 | | – | 5 | – | 1 | – | 3 | 4 | 8 | 63 | 82 | 15 | – | – | – | – | – | – | – | – | – | |
|  | Cum. % | | – | | – | | – | | – | | – | | – | | – | | 3.7 | | – | | – | | 5.3 | | – | 7.8 | – | 8.3 | – | 9.9 | 12 | 16.2 | 49.2 | 92.1 | 100 | – | – | – | – | – | – | – | – | – | |
|  | % | | – | | – | | – | | – | | – | | – | | – | | 3.7 | | – | | – | | 1.6 | | – | 2.5 | – | 0.5 | – | 1.6 | 2.1 | 4.2 | 33.0 | 42.9 | 7.9 | – | – | – | – | – | – | – | – | – | |
| CTB | N | | – | | – | | – | | – | | – | | 7 | | – | | – | | – | | – | | 1 | | – | 6 | – | 1 | – | 3 | 2 | – | 5 | 21 | 145 | – | – | – | – | – | – | – | – | – | |
|  | Cum. % | | – | | – | | – | | – | | – | | 3.7 | | – | | – | | – | | – | | 4.2 | | – | 7.3 | – | 7.8 | – | 9.4 | 10.4 | – | 13 | 24 | 100 | – | – | – | – | – | – | – | – | – | |
|  | % | | – | | – | | – | | – | | – | | 3.7 | | – | | – | | – | | – | | 0.5 | | – | 3.1 | – | 0.5 | – | 1.6 | 1.0 | – | 2.6 | 11.0 | 75.9 | – | – | – | – | – | – | – | – | – | |
| CRO | N | | 4 | | – | | 3 | | – | | 8 | | – | | 2 | | – | | 2 | | – | | 2 | | – | 14 | – | 50 | – | 101 | 5 | – | – | – | – | – | – | – | – | – | – | – | – | – | |
|  | Cum. % | | 2.1 | | – | | 3.7 | | – | | 7.9 | | – | | 8.9 | | – | | 9.9 | | – | | 10.9 | | – | 18.2 | – | 44.4 | – | 97.3 | 100 | – | – | – | – | – | – | – | – | – | – | – | – | – | |
|  | % | | 2.1 | | – | | 1.6 | | – | | 4.2 | | – | | 1.0 | | – | | 1.0 | | – | | 1.0 | | – | 7.3 | – | 26.2 | – | 52.9 | 2.6 | – | – | – | – | – | – | – | – | – | – | – | – | – | |
| CXM | N | | – | | – | | – | | – | | – | | – | | – | | – | | – | | 6 | | – | | – | 1 | – | 1 | – | 1 | 13 | 14 | 38 | 46 | – | 21 | – | 27 | 23 | – | – | – | – | – | |
|  | Cum. % | | – | | – | | – | | – | | – | | – | | – | | – | | – | | 3.1 | | – | | – | 3.6 | – | 4.1 | – | 4.6 | 11.4 | 18.7 | 38.6 | 62.7 | – | 73.7 | – | 87.8 | 100 | – | – | – | – | – | |
|  | % | | – | | – | | – | | – | | – | | – | | – | | – | | – | | 3.1 | | – | | – | 0.5 | – | 0.5 | – | 0.5 | 6.8 | 7.3 | 19.9 | 24.1 | – | 11.0 | – | 14.1 | 12.0 | – | – | – | – | – | |
| CLR | N | | – | | – | | – | | – | | – | | – | | – | | – | | – | | – | | – | | – | – | – | – | 7 | – | 4 | 2 | 3 | 59 | – | 82 | – | 18 | – | 3 | 13 | – | – | – | |
|  | Cum. % | | – | | – | | – | | – | | – | | – | | – | | – | | – | | – | | – | | – | – | – | – | 3.7 | – | 5.8 | 6.8 | 8.4 | 39.3 | – | 82.2 | – | 91.6 | – | 93.2 | 100 | – | – | – | |
|  | % | | – | | – | | – | | – | | – | | – | | – | | – | | – | | – | | – | | – | – | – | – | 3.7 | – | 2.1 | 1.0 | 1.6 | 30.9 | – | 42.9 | – | 9.4 | – | 1.6 | 6.8 | – | – | – | |
| LVX | N | | – | | – | | – | | 8 | | – | | – | | 9 | | – | | 84 | | – | | 12 | | – | 7 | – | 19 | – | 4 | 18 | 7 | 2 | 1 | – | 14 | 6 | – | – | – | – | – | – | – | |
|  | Cum. % | | – | | – | | – | | 4.2 | | – | | – | | 8.9 | | – | | 52.9 | | – | | 59.2 | | – | 62.9 | – | 72.8 | – | 74.9 | 84.3 | 88.0 | 89.0 | 89.5 | – | 96.8 | 100 | – | – | – | – | – | – | – | |
|  | % | | – | | – | | – | | 4.2 | | – | | – | | 4.7 | | – | | 44.0 | | – | | 6.3 | | – | 3.7 | – | 9.9 | – | 2.1 | 9.4 | 3.7 | 1.0 | 0.5 | – | 7.3 | 3.1 | – | – | – | – | – | – | – | |
| MXF | N | | – | | – | | – | | 8 | | – | | – | | 8 | | – | | 59 | | – | | 35 | | – | 10 | – | 13 | – | 9 | 15 | 11 | 2 | 9 | – | 8 | 4 | – | – | – | – | – | – | – | |
|  | Cum. % | | – | | – | | – | | 4.2 | | – | | – | | 8.4 | | – | | 39.3 | | – | | 57.6 | | – | 62.8 | – | 69.6 | – | 73.8 | 81.7 | 87.5 | 88.5 | 93.2 | – | 97.4 | 100 | – | – | – | – | – | – | – | |
|  | % | | – | | – | | – | | 4.2 | | – | | – | | 4.2 | | – | | 30.9 | | – | | 18.3 | | – | 5.2 | – | 6.8 | – | 4.2 | 7.9 | 5.8 | 1.0 | 4.7 | – | 4.2 | 2.1 | – | – | – | – | – | – | – | |
| TET | N | | – | | – | | – | | – | | – | | – | | – | | – | | – | | – | | – | | – | – | 15 | – | – | 106 | 35 | – | 3 | 3 | – | 7 | – | 5 | – | 8 | 9 | – | – | – | |
|  | Cum. % | | – | | – | | – | | – | | – | | – | | – | | – | | – | | – | | – | | – | – | 7.9 | – | – | 63.4 | 81.7 | – | 83.3 | 84.9 | – | 88.6 | – | 91.2 | – | 95.4 | 100 | – | – | – | |
|  | % | | – | | – | | – | | – | | – | | – | | – | | – | | – | | – | | – | | – | – | 7.9 | – | – | 55.5 | 18.3 | – | 1.6 | 1.6 | – | 3.7 | – | 2.6 | – | 4.2 | 4.7 | – | – | – | |
| SXT | N | | – | | – | | – | | – | | – | | 9 | | – | | – | | 2 | | – | | 9 | | – | 13 | – | 5 | – | 1 | – | – | – | 19 | – | 43 | 90 | – | – | – | – | – | – | – | |
|  | Cum. % | | – | | – | | – | | – | | – | | 4.7 | | – | | – | | 5.7 | | – | | 10.4 | | – | 17.2 | – | 19.8 | – | 20.3 | – | – | – | 30.2 | – | 52.7 | 100 | – | – | – | – | – | – | – | |
|  | % | | – | | – | | – | | – | | – | | 4.7 | | – | | – | | 1.0 | | – | | 4.7 | | – | 6.8 | – | 2.6 | – | 0.5 | – | – | – | 9.9 | – | 22.5 | 47.1 | – | – | – | – | – | – | – | |

–, not applicable; AMC, amoxicillin/clavulanic acid; AMP, ampicillin; AMX, amoxicillin; AZM, azithromycin; CDR, cefdinir; CEC, cefaclor; CFM, cefixime; CLR, clarithromycin; CPD, cefpodoxime; CRO, ceftriaxone; CTB, ceftibuten; CTX, cefotaxime; Cum., cumulative; CXM, cefuroxime; LVX, levofloxacin; MXF, moxifloxacin; SXT, trimethoprim/sulfamethoxazole; TET, tetracycline.

Bold vertical bars in table correspond to the CLSI-susceptible breakpoints.
